# Efficient Aflatoxin B1 Sequestration by Yeast Cell Wall Extract and Hydrated Sodium Calcium Aluminosilicate Evaluated Using a Multimodal In-Vitro and Ex-Vivo Methodology

**DOI:** 10.3390/toxins13010024

**Published:** 2021-01-01

**Authors:** Alexandros Yiannikouris, Juha Apajalahti, Hannele Kettunen, Suvi Ojanperä, Andrew N. W. Bell, Jason D. Keegan, Colm A. Moran

**Affiliations:** 1 Chemistry and Toxicology Division, Center for Animal Nutrigenomic and Applied Animal Nutrition, Alltech Inc., 3031 Nicholasville, KY 40356, USA; 2 Alimetrics Ltd., Koskelontie 19B, 02920 Espoo, Finland; j.apajalahti@alimetrics.com (J.A.); hakettunen@hotmail.com (H.K.); suvi.ojanpera@gmail.com (S.O.); 3 Alltech Ireland, Sarney, Summerhill Road, A86 X006 Dunboyne, Ireland; andrew.bell@alltech.com (A.N.W.B.); jasondjdk@gmail.com (J.D.K.); 4 Alltech SARL (France), ZA La Papillionnière, Rue Charles Amand, 14500 Vire, France; cmoran@alltech.com

**Keywords:** aflatoxin B1, mycotoxin, yeast cell wall extract, adsorption, HSCAS, sequestration, absorption, bioavailability, in vitro, ex vivo

## Abstract

In this work, adsorption of the carcinogenic mycotoxin aflatoxin B1 (AFB1) by two sequestrants—a yeast cell wall-based adsorbent (YCW) and a hydrated sodium calcium aluminosilicate (HSCAS)—was studied across four laboratory models: (1) an in vitro model from a reference method was employed to quantify the sorption capabilities of both sequestrants under buffer conditions at two pH values using liquid chromatography with fluorescence detection (LC-FLD); (2) in a second in vitro model, the influence of the upper gastrointestinal environment on the mycotoxin sorption capacity of the same two sequestrants was studied using a chronic AFB1 level commonly encountered in the field (10 µg/L and in the presence of feed); (3) the third model used a novel ex vivo approach to measure the absorption of ^3^H-labelled AFB1 in the intestinal tissue and the ability of the sequestrants to offset this process; and (4) a second previously developed ex vivo model readapted to AFB1 was used to measure the transfer of ^3^H-labelled AFB1 through live intestinal tissue, and the influence of sequestrants on its bioavailability by means of an Ussing chamber system. Despite some sorption effects caused by the feed itself studied in the second model, both in vitro models established that the adsorption capacity of both YCW and HSCAS is promoted at a low acidic pH. Ex vivo Models 3 and 4 showed that the same tested material formed a protective barrier on the epithelial mucosa and that they significantly reduced the transfer of AFB1 through live intestinal tissue. The results indicate that, by reducing the transmembrane transfer rate and reducing over 60% of the concentration of free AFB1, both products are able to significantly limit the bioavailability of AFB1. Moreover, there were limited differences between YCW and HSCAS in their sorption capacities. The inclusion of YCW in the dietary ration could have a positive influence in reducing AFB1′s physiological bioavailability.

## 1. Introduction

Aflatoxins have been found to contaminate a variety of agricultural products, such as maize and other small-grain cereal crops, both pre-harvest and during storage. *Aspergillus* spp. within the section *Flavi* are principal producers of aflatoxins [1], fungal secondary metabolites (mycotoxins) that include aflatoxin B1 (AFB1), aflatoxin B2, aflatoxin G1, and aflatoxin G2 [2]. While these are regarded as the four most significant mycotoxins in the class [3], AFB1 is the most potent of the group, and the effects of its consumption have been well characterised [4]. AFB1 is passively absorbed through the gastrointestinal mucosa at a very high rate [5,6] and, due to its lipophilic nature, is characteristically sequestered in the liver. In the liver, AFB1 is converted to the highly reactive 8,9-epoxide-AFB1 (AFB0) primarily through the cytochrome P450 oxidative system [7]. AFB0 is responsible for most of the toxicity of AFB1, forming adducts with DNA (AFB1 N7-guanine) and proteins (AFB1 Lys) within the cell, leading to significant macromolecular disruption [4,8].

Acute aflatoxicosis, a comparatively rare phenomenon, has been shown to stem from impaired mitochondrial function and the resulting oxidative stress and disrupted lipid metabolism [9]. Through chronic exposure, AFB1 and aflatoxin M1 (AFM1, a phase I metabolite issued from the hydroxylation of AFB1) have shown teratogenic, immunotoxic, mutagenic, and hepatotoxic activity and are classified as Group I carcinogens [9,10,11]. Accordingly, these aflatoxins have been implicated in the causation of hepatocellular carcinoma [8] and extrahepatic carcinomas in both humans and animals [12].

While commonly associated with non-industrialised countries, aflatoxins continue to be detected in grain products and animal by-products such as milk, meat, and eggs in developed nations, a significant issue given the widespread consumption of these foods by humans both young and old [3,13] and especially in places where access to safe food remains a significant challenge. Tolerance limits are set for many countries to mitigate much of the risk posed by the presence of mycotoxins in food and feed. Applied to animal feedstuffs in the United States, AFB1 limits range from 20 to 300 µg total aflatoxins per kg of feed material (depending on the production stage of the animal and the feed material), with a final 20 µg/kg tolerance applied to human food [14], while the European Union stipulates comparatively tighter limits with 20 µg/kg for feed materials, 10 µg/kg for all complimentary and complete feeds, and as low as 5 µg/kg for any compound feed destined for dairy-producing or young animals [15].

While active strategies are followed to control mycotoxin contamination in the field or during storage by implementing good agricultural practices, and despite employing chemical and/or mechanical means of reducing overall contamination (sorting, dehulling, washing, ozonation, or ammonification to chemically degrade existing mycotoxins), mycotoxin contamination is unavoidable [16]. This task is forecast to become more difficult as we face changes in global climatic conditions and/or extreme weather patterns that stand to favour mycotoxin-producing organisms [17], further complicated by the international trading of feed ingredients and increasing the chances of unattended patterns of contamination. While acknowledging the procedural limitations to the accurate quantification of mycotoxins in bulk feedstuffs (most often due to sampling issues rather than analytical imprecision, e.g., heterogenous distribution within a lot and/or limited sampling plans), many countries have implemented standardised methods to moderate these limitations as much as is reasonably practical [18,19].

Following a reduction in mycotoxin levels in feedstuffs to within acceptable tolerance, the final mitigative step should focus on reducing the bioavailability of mycotoxins to digestive absorption and decrease their interactions with animal organisms, thereby minimising potential low-dose chronic effects that could affect animal performance. The inclusion of adsorbent materials in animal feed is perhaps the most economically viable means of achieving this “final-stage-control” of low-level or unaccounted for contamination [20]. Such supplementation can lead to the sequestration or adsorption of mycotoxins, thereby limiting the concentrations available to the animal until being excreted back into the environment [21,22] and/or promoting overall gut health, offsetting some of the related deleterious toxic effects [23].

While significant bodies of work have demonstrated the sequestration of AFB1 under a range of sorbents, many of the investigations focused on efficacy in elevated mycotoxin levels with minimal evaluations in the context of the digestive system. Appropriately, fewer studies have been carried out at low, chronic, or regulation-comparable levels. The aim of this study was to improve our understanding of the efficacy and mechanisms of the protection afforded to biological systems by a yeast cell wall-based sorbent (YCW, Mycosorb^®^) and a hydrated sodium calcium aluminosilicate (HSCAS) clay at comparatively low AFB1 concentrations. To achieve this, two individual in vitro and two individual ex vivo models were utilised. The first model (Model 1) is an industry standard method proposed by the FEFANA (the EU Association of Specialty Feed Ingredients and their Mixtures) for in vitro efficacy testing of mycotoxin binders [24]. The second developed in vitro model (Model 2) was employed to assess the sequestrant sorption capabilities under physiologically relevant conditions and at low (10 µg/L) AFB1 concentrations. A third model (Model 3) was used to measure the ex vivo absorption of AFB1 into live intestinal tissue and the degree to which the sequestrants could help offset such a mechanism and, finally, a fourth model, based on earlier work [25] (Model 4), was used again to verify the progress in ex vivo AFB1 absorption across living intestinal tissue and determine the sequestrant’s impact on the toxin bioavailability in this system.

## 2. Results

### 2.1. Model 1: In Vitro (FEFANA) Protocol for Assessing AFB1 Sequestration

**Figure 1:**
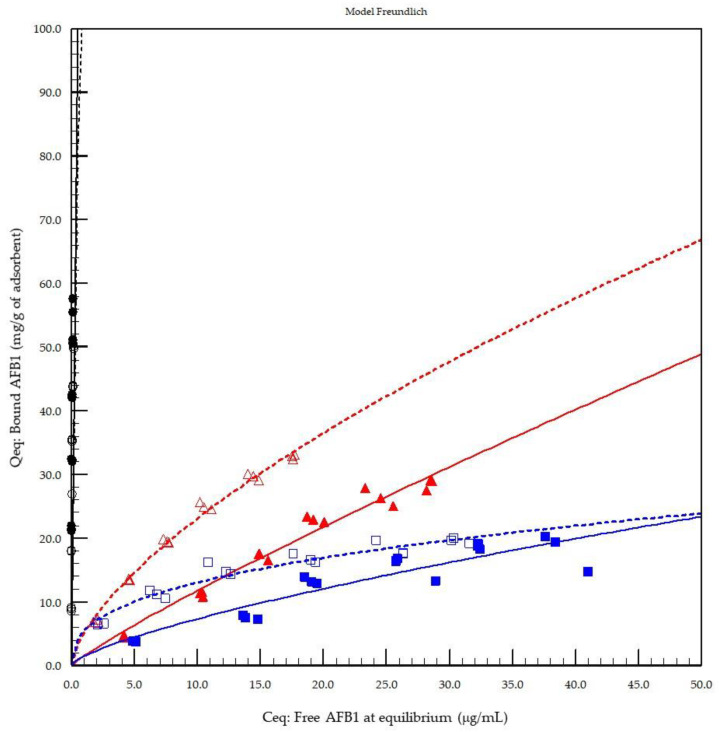
Concentrations of bound vs. free aflatoxin B1 (AFB1) evaluated at pH 3.0 and 7.0 using either a yeast cell wall-based adsorbent (YCW) or hydrated sodium calcium aluminosilicate (HSCAS) compared with an activated carbon (AC) reference material. The concentration of free AFB1 at equilibrium is expressed in µg/mL, and the corresponding bound concentration of AFB1 is expressed in mg/g for each adsorbent used: AC (black circles), YCW (blue squares), and HSCAS (red triangles) at pH 3.0 (open markers and dashed lines) and pH 7.0 (filled markers and solid lines). All replicate values are displayed in the graphic. Adsorption curves were fitted using the least mean squares method to match the Freundlich equation (Qeq = Kf C1/*n* where Qeq is the y-axis, and Ceq is the *x*-axis).

The plot presented in Figure 1 represents the regression obtained using the Freundlich equation. Hill’s equation with *n* sites was particularly suitable for YCW (*R*^2^ = 0.9591) and HSCAS (*R*^2^ = 0.9948) at pH 3.0 and was the best-fitting model for YCW at pH 7.0 (*R*^2^ = 0.8929) but provided results less adapted for the activated carbon reference material. The activated carbon response provided poor regression results even when fitted with the Freundlich equation (*R*^2^ = 0.6608) at pH 3.0 and could not be fitted with any of the models used at pH 7.0. According to Freundlich data fitting, the adsorption capacity (Kf) and intensity were comparable for both YCW and HSCAS (Table 1), with higher capacity and intensity coefficient observed for YCW at pH 3.0 than for HSCAS and higher capacity for HSCAS but a lower intensity compared with YCW at pH 7.0.

The percentage of AFB1 bound to activated carbon exceeded 99.8% in the gamut of concentrations tested for AFB1 (Table 2). Both test materials (0.1% YCW and 0.1% HSCAS) demonstrated effective binding of AFB1 across the concentration range at both pH levels. At the lowest AFB1 dosage rate (10 µg/mL), YCW and HSCAS effectively adsorbed 74% and 78% of the toxins, respectively, under acidic conditions, but the adsorption rate fell to 42% and 52% for both the adsorbents in the neutral condition. At pH 3.0, as the AFB1 concentration increased in the reaction vessel, the relative proportion of bound AFB1 decreased for both adsorbents, indicating some degree of saturation that was more pronounced for YCW. The overall average adsorption rate was 71% for the tested HSCAS and significantly differed (*p* < 0.01) from that of the YCW, with 53% overall adsorption, but with a high coefficient of variation (25%) for the latter due to the saturation effect at the highest dose of AFB1 tested. The trend at pH 7.0 differed from that at pH 3.0. At pH 7.0, regardless of the initial dosage rate, YCW sequestered an average of 37%, while the HSCAS exhibited a stable adsorption of around 52% that of the AFB1. The AFB1 concentration increase had little significant impact on the bound proportion, indicating a difference in saturation at pH 7.0 compared with pH 3.0. These results support the assertion that reduced pH tends to promote the adsorption of AFB1, whereas at a neutral pH, binding is influenced by pressure exerted from the concentration of free toxins.

### 2.2. Model 2: In Vitro Assessment of AFB1 Binding in Simulated Stomach Conditions

**Figure 2:**
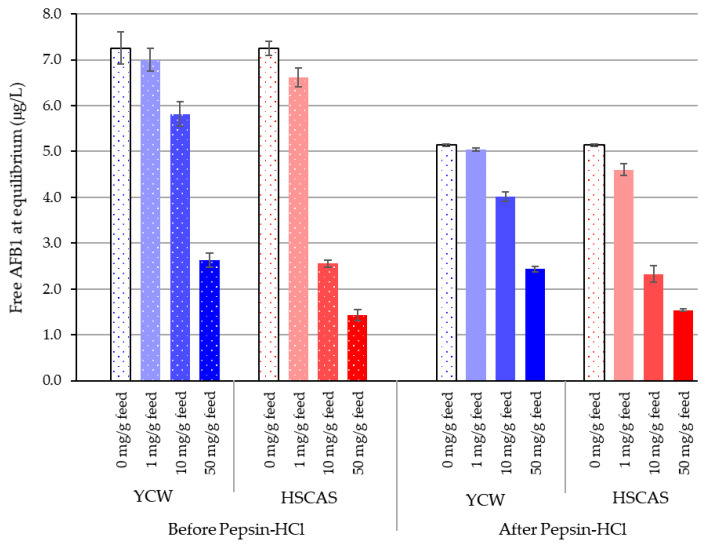
Effect of the presence of feed before (dotted bars) and after (solid bars) digestive treatments (with pepsin–HCl) on the concentration of free aflatoxin B1 (AFB1, μg/L) present in the reaction environment and with or without (white bars) three inclusion levels (expressed in mg/g of feed) of either a yeast cell wall-based adsorbent (YCW, blue bars) or a hydrated sodium calcium aluminosilicate (HSCAS, red bars).

Simulated stomach conditions were used in a second model to simulate the physiological conditions of the use of the tested product and its impact on the concentration of free AFB1 of YCW or HSCAS admixed with a feed material at four inclusion levels (Figure 2). Free AFB1 was stabilised at 7.25 µg/L in the presence of 6.6% *w*/*v* feed material following 2 h incubation in water and reduced to 5.14 µg/L after 2 h of incubation with pepsin–HCl in the absence of adsorbent. The addition of 0.1% YCW further reduced the free AFB1 concentration from 7.25 to 7.00 µg/L after 2 h incubation in water at 37 °C, dropping to 5.04 µg/L after 2 h of incubation with pepsin–HCl. HSCAS inclusion at 0.1%, 1.0%, and 5.0% resulted in a significant decrease (*p* < 0.05) in free AFB1 in the reaction media with pepsin–HCl treatment compared with incubation of the AFB1 with the feed alone. Identically, YCW inclusion at 1.0% and 5.0%, but not 0.1%, significantly reduced the amount of free AFB1 (Table 3). Significant differences were observed when comparing the levels of inclusion of 1.0% and 5.0% for YCW and HSCAS. The maximal reduction in AFB1 was around 76% and 86%, respectively, with HSCAS showing a higher decrease in free AFB1 than YCW. Interestingly, the addition of feed alone in the reaction environment in the presence of water or after pepsin–HCl treatment also reduced the availability of free AFB1 by a proportion of 28% and almost 49%, respectively, showing AFB1′s potential interaction with the feed matrix. A comparison of the AFB1 sequestration capabilities of the test materials prior to and following pepsin–HCl treatment revealed a 29% increase in binding attributable to feed, a 28–31% increase in binding in the presence of YCW (0.1% and 1%, respectively), and a 30% increase in binding in the presence of HSCAS (0.1%). This indicates significant pH-mediated facilitation of sequestration at a low AFB1 concentration.

### 2.3. Model 3: Ex Vivo Demonstration of Binding Efficiency in the Lower Gastro-Intestinal Tract

Model 3 was designed to investigate the ability of AFB1 to accumulate ex vivo in the live proximal jejunal tissue of a rat intestine maintained at 37 °C in the presence or absence of each of the two tested materials, YCW and HSCAS. A pre-study was performed to determine the suitable time of incubation and the concentration of AFB1 needed to observe incremental tissue accumulation. A final AFB1 concentration of 10 µg/L was selected and spiked with 0.15 μCi/mL of ^3^H-labelled AFB1 for quantification purposes. It was determined that the linearity of the accumulation of AFB1 was suitable over a 4 min period, beyond which it tended to level off (as tested in a pre-study up to 8 min). The proportion of AFB1 accumulation was found to be identical when 1.0 µg/L of AFB1 was tested, confirming that the uptake was not achieved with the chosen AFB1 concentration, due to saturation but rather than due to the model limits.

**Figure 3:**
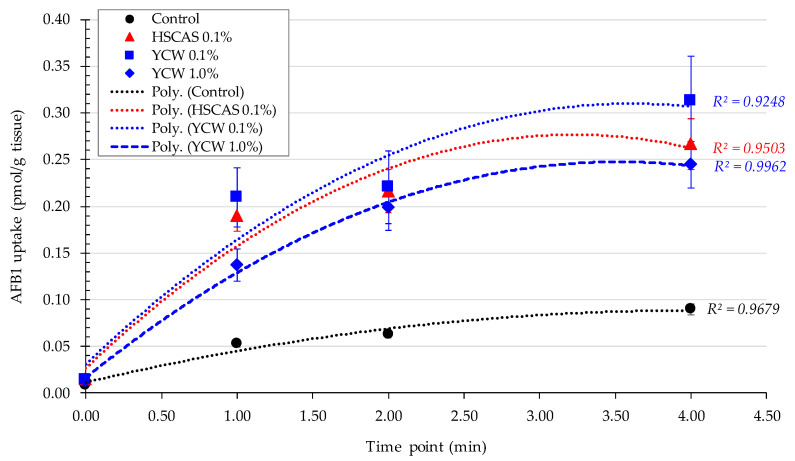
Aflatoxin B1 (AFB1) uptake (µmol/g of live intestinal tissue) by the proximal jejunum tissue as a function of the incubation time (min) with or without (Control, Ctrl) the addition of either a yeast cell wall-based adsorbent (YCW) or hydrated sodium calcium aluminosilicate (HSCAS) at different inclusion rates in the environment of the test. Error bars represent the standard error of the mean. Polynomial regression of the second order was used to fit the experimental points.

The trends seen in Figure 3 were statistically compared, as outlined in Table 4. These results show that over the 4 min incubation time, 2.5 to 3.5 times more AFB1 was accumulated on the tissue slices when the adsorbent was present compared with the control. Although there was a significant increase in AFB1 sequestered by the tissue (µmol/g tissue) when comparing the untreated (control) tissue to the treated tissue slices, no significant difference was observed between the YCW and HSCAS test material investigations, nor were different inclusion rates observed for YCW.

The time-dependent nature of the binding intensity is illustrated in Figure 3. The application of linear regression allowed determination of the absorption coefficient, as displayed in Table 5. The slope coefficients for both YCW treatments differed only marginally, with 0.0359 µmol/g tissue/min and 0.0339 µmol/g tissue/min for 0.1% and 1.0% YCW, respectively. While the slope produced by 0.1% HSCAS (0.0255 µmol/g tissue/min) differed from that of YCW, the difference was not statistically significant.

Contrary to expectations (Figure 3), the measured quantities of AFB1 appeared to increase in the presence of the tested products, as determined by visual observation of the adhesion of adsorbents to the intestinal tissue. The detection of AFB1 in the tissue could thus be attributed not to an uptake mechanism but rather to the presence of AFB1 as a complex with the adsorbent material on the tissue surface, which was still available for quantification by liquid scintillation counting.

**Figure 4:**
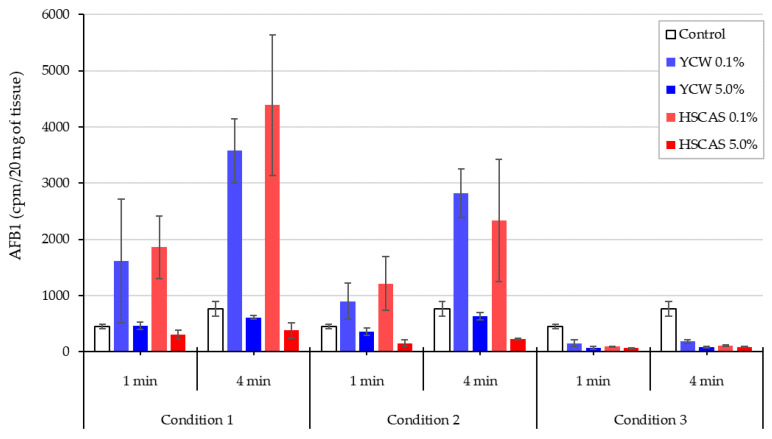
Aflatoxin B1 (AFB1) relative uptake due to non-specific surface interaction, expressed in cpm/20 mg (or rather pmol/g) of live intestinal tissue (proximal jejunum) over two incubation periods (1 and 4 min) with or without (Control) the addition of either a yeast cell wall-based adsorbent (YCW) or hydrated sodium calcium aluminosilicate (HSCAS), each tested at two different inclusion rates (0.1%, 5.0%, *w*/*v*). Three experimental conditions were tested—a standard incubation treatment with AFB1, with the adsorbent and intestinal tissue all at once (Condition 1); a procedure involving pre-incubation of the adsorbent and toxin beforehand followed by incubation with intestinal tissue (Condition 2); and a two-step procedure using Condition 2 followed by separation via centrifugation of the insoluble test product prior to incubation of the supernatant with intestinal tissue (Condition 3). Error bars represent the standard deviation.

This result was confirmed using a two-step experimental setup, where the AFB1–adorbent interaction was performed first, followed by a centrifugation step to remove the complexed AFB1 and test material (Figure 4, Condition 3). The supernatant containing the residual free AFB1 was then applied to the intestinal tissue; in this instance, only a minimal quantity of AFB1 was seen to be taken up by the tissue, which was indicative of low AFB1 bioavailability compared to the adsorbent-free control. This contrasts with the significant AFB1–tissue interactions when all materials (AFB1, adsorbent, and intestinal tissue) are incubated together (Condition 1) or when the absorbent has been pre-incubated with AFB1 before this mixture is incubated with tissue slices (Condition 2), revealing an important experimental bias.

### 2.4. Model 4: Impact of Adsorption Capacity on Transmission of AFB1 through Live Intestinal Tissue

**Figure 5:**
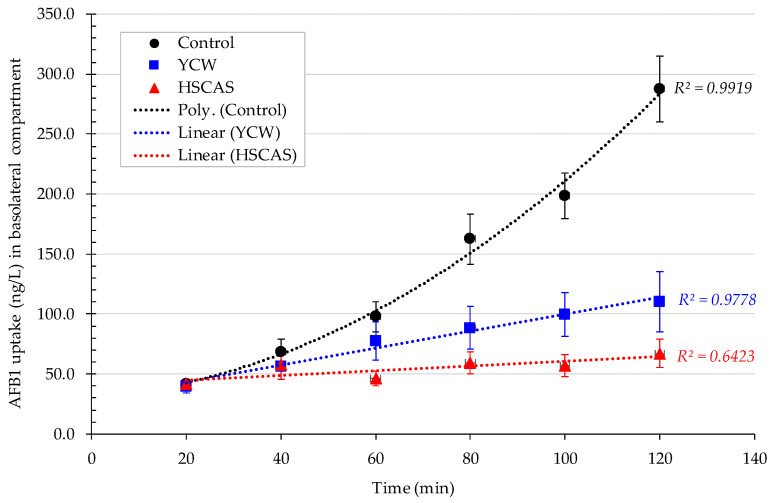
Aflatoxin B1 (AFB1) uptake (ng/L) through live rat intestinal tissue isolated from 10 rats measured with two replicates each and quantification of AFB1 in the basolateral compartment of a bicameral Ussing chamber system after the addition of a total concentration of 30 µg/L of AFB1 (^3^H-AFB1) in the apical chamber with or without (Control) the addition of either a yeast cell wall-based adsorbent (YCW) or hydrated sodium calcium aluminosilicate (HSCAS) over a 120 min kinetic period. Regression for the control was best fit with polynomial regression of the second order, while polynomial regression of the first order (linear) was used in the case of HSCAS and YCW. Error bars represent the standard error of the mean.

The fourth model was applied using an Ussing bicameral experimental system setup to account for the digestive absorption process by measuring the concentration of AFB1 (^3^H activity) in the apical compartment corresponding to the lumen of the digestive tract, the basolateral compartment corresponding to the systemic circulation, and the intestinal tissue following the addition of AFB1 in the apical chamber without (Control) or in the presence of YCW or HSCAS used at an inclusion rate of 0.3% *w*/*v*. By examining the AFB1 uptake and transport to the basolateral compartment (Figure 5) without the use of any adsorbent, we observed an increase in the AFB1 concentration over the 2 h time course of the kinetics. The transport kinetics of AFB1 alone followed a second order polynomial regression, while the addition of either adsorbent made the AFB1 transfer kinetics more linear. The addition of YCW or HSCAS significantly decreased the transfer of AFB1 to the basolateral compartment over the same period of time, respectively, yielding a mean concentration of 109.9 and 67.2 ng/L compared with an AFB1 transfer rate of 287.9 ng/L when no adsorbent was used. The effect of the sequestrant in reducing the transfer rate of AFB1 was statistically significant after 60 min (*p* < 0.01) for the HSCAS and 80 min for YCW (*p* < 0.05). The final concentration of AFB1 measured in the basolateral compartment did not differ significantly between the YCW and HSCAS treatments, demonstrating their effectiveness in significantly limiting the transfer rate of AFB1 through the intestinal tissue.

The application of linear regression on this dataset (assuming a first order polynomial model for the control) gave slope values of 0.71 ng/L/min for 0.3% YCW and 0.2 ng/L/min for 0.3% HSCAS, while the slope of the control was significantly higher at 2.39 ng/L/min. Further statistical evaluation of the regression (Table 6) with 95% confidence intervals demonstrated a marked interspersion of the values obtained for YCW and HSCAS, whereas a clear segregation of these sets of values was obtained from the control.

**Figure 6:**
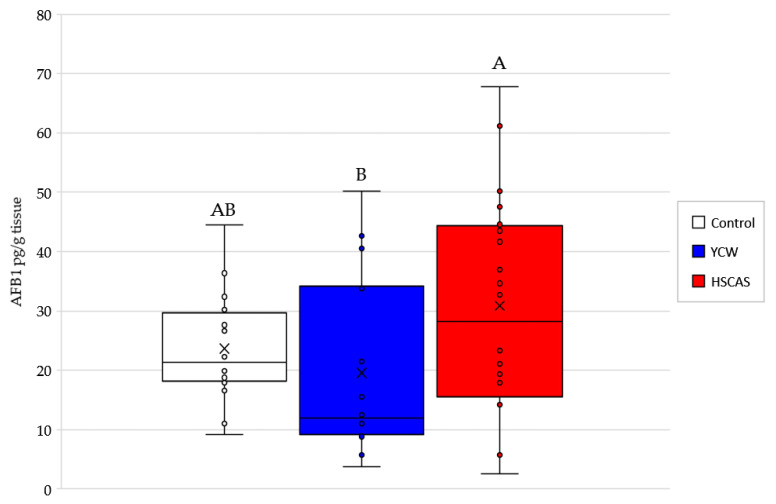
Box plot of the aflatoxin B1 (AFB1) accumulation expressed in pg/g of tissue in the rat intestinal tissue after 120 min incubation in an Ussing Chamber ex vivo experiment using 30 µg/L of AFB1 (^3^H-AFB1) added to the apical chamber with or without (Control) the addition of either a yeast cell wall-based adsorbent (YCW) or hydrated sodium calcium aluminosilicate (HSCAS). Different superscript letters above the bars indicate significant differences between treatments, *p* < 0.05.

Studying the accumulation of AFB1 in intestinal tissue (Figure 6) at the end of the experimental period in the Ussing chamber ex vivo system revealed that the tissue accumulation of AFB1 remained around ~28 pg/g tissue, regardless of the presence or absence of an adsorbent. Statistically significant (*p* = 0.036) differences were found between YCW and HSCAS, which showed lower AFB1 accumulation in the former compared to the latter. No statistical difference, however, was observed between the adsorbent treatments and the control.

## 3. Discussion

The first model of the in vitro adsorption evaluation demonstrated that activated carbon played the role of a reference material with the strongest AFB1 binding capacity at both pH 3.0 (stomach equivalent) and pH 7.0 (intestine equivalent) compared to the other test products (Figure 1). The results from the Freundlich model were compared to those of the Langmuir and Hill’s models with *n* site equations, which are other models recommended by FEFANA for the evaluation of adsorbents. The Freundlich model, although not necessarily the model providing the best fit, was able to characterise all adsorbents at the two different tested pH values (Table 1). Hill’s equation [26] with *n* sites was particularly well-adapted to the evaluation of YCW, confirming the appropriateness of the model for organic adsorbents, as demonstrated previously [25]; however, the model was less well-adapted to the AC reference material in the particular case of AFB1. All models tested were unable to correctly fit the reference material due to the lack of a defined experimental saturation phase with the concentrations tested. However, Freundlich was selected as an all-round model for comparing the other tested sorbent materials of a mixed nature, such as inorganic binders (HSCAS) and organic binders (YCW), thus confirming the previously observed results obtained with the same model applied to zearalenone [25]. Interestingly, when the cooperativity factor (*n* sites) of Hill’s model was examined, the results revealed that HSCAS lacks cooperativity, as expected, with a factor around a value of 1 (0.96), whereas YCW exhibited <1 at pH 3.0 (0.76) and >1 at pH 7.0 (1.63), accounting for the dynamicity of their interactions due to the flexibility and spatial reorganisation capacity of the parietal carbohydrate fraction of the yeast compared to the very static HSCAS physical organisation, thereby providing a restrictive and rigid site for interaction.

The key desirable element for in vivo mycotoxin binding is maximising the adsorption at a low pH where mycotoxins are more freely available in the digestate (chyme) and retaining the maximum quantity of bound toxins (minimising desorption) when conditions become less favourable (as pH increases in the gut). This desorption of mycotoxins when the pH increases from acidic stomach conditions to neutral/basic intestinal conditions is well established [27,28,29,30]. The larger difference observed between the pH tested in the first model and that of the HSCAS may be attributable to the greater cation exchange capacity of the latter material [31], which is more susceptible to promotion and demotion by other ionic sources and is more greatly influenced by the pH. Conversely, the smaller differences between pH measurements observed with YCW could be attributed to the complex structure of the yeast cell wall involving non-charged polymers of glucose and mannose, which are less likely to be influenced by pH changes in their sequestration activity and, as such, can better retain bound mycotoxins during pH transitions [27].

We acknowledge that the AFB1 concentrations in the proposed FEFANA model were markedly higher than the actual permissible levels in animal feeds. The lowest concentration used here was two hundred times higher than the recommended level for pharmacokinetic studies (50 µg/kg of feed). The purpose of this model was not to demonstrate its efficacy in the presence of low mycotoxin concentrations but to establish the degree to which the test materials would bind the target mycotoxin. Such trends were innately more evident at higher AFB1 concentrations, and the experimental fluctuations were commensurately lower. That being said, this model does not factor in the complexities and nuances of mycotoxin binding in vivo, and extrapolation of the binding efficacy at these particularly high concentrations to applications in live models would be tenuous at best. These limitations led to the development and application of novel experimental setups (Models 2–4) to more elegantly and realistically simulate the conditions of AFB1 contamination encountered, especially in animal production, thus providing a deeper understanding of the observable effects ex vivo and allowing more confident real-world inference.

Having established that the test materials can effectively bind AFB1 at a low pH, Model 2 was designed to build upon Model 1 and demonstrate the sequestration of AFB1 at the low “chronic” levels one might expect to find in feedstuffs, using simulated upper gastrointestinal (GI) conditions. The results suggest an innately similar promotion of toxin–binder interactions with the organic materials (feed and YCW) under pepsin–HCl treatment. An inclusion of 0.1% HSCAS reduced the free AFB1 concentration from 7.25 to 6.62 µg/L in water and to 4.60 µg/L after pepsin–HCl digestion, likely due to the enhanced ionisation of clay molecules in this environment. The results obtained for HSCAS at 0.1% were not significantly different from those obtained with YCW (*p* < 0.05). An increase in the inclusion of HSCAS significantly enhanced the ability of HSCAS to sequester more AFB1 than YCW. Nevertheless, the YCW feed mixture was also able to significantly reduce free AFB1 down to 2.6 and 2.4 µg/L from the initial 10 µg/L added to the reaction environment. The biggest difference between YCW and HSCAS was noted at an inclusion of 1.0% *w*/*v* of the tested material (Table 3). This could be explained by the differences not only in the types of chemical interactions generated, but also because of different optima of sequestration activities that are to be expected from the differences in the composition of the two tested materials.

Interestingly, the observation that the feed can affect the availability of free AFB1 confirmed the existence of potential interactions between the toxins and the organic matrix of the feed, which is also composed of carbohydrates but of a different nature than that of the YCW. In a pre-study (data not shown), the inclusion of 3.3%, 10%, and 30% feed to water (*w*/*v*) was found to decrease the free AFB1 in the reaction media from 10 down to 8.9, 7.6, and 5.9 µg/L, with a respective decrease of 11.1%, 23.6%, and 41.1% of the free AFB1 after 2 h of incubation at 37 °C. In the presence of pepsin–HCl, the reduction in free AFB1 was further pronounced, with concentrations down to 7.8, 5.8, and 4.4 μg/L, representing a reduction of 22.2%, 42.3%, and 56.2%, respectively, in free AFB1. Activated vegetable fibre (AVF) is successfully used in industrial processes like brewing to systematically reduce the concentrations of mycotoxins and agricultural chemicals to mitigate their impact on waste materials often recycled into animal feed [32]. The AVF for this application is often derived from micronized wheat envelopes (bran) comprised of a variety of carbohydrates—mainly hemicellulose molecules—composed of β-(1,4)-linked-glucose, -mannose, and -galactose [33]. However, we hypothesise that the degradation of these molecules in the hindgut is sometimes assisted with enzymatic aids such as β-d-glucanase but tends to liberate the mycotoxins that have interacted with this vegetal carbohydrate fraction that is then further absorbed as an energy source for the host [34]. This degradation progressively reduces the interactions, resulting in the release of bound mycotoxins, making them bioavailable to the host once again and thus counterbalancing the competition between the feed matrix and the adsorbent for access to the mycotoxin. YCW consists of an insoluble carbohydrate fraction specifically made out of long chains of β-(1,3)-d-glucans branched with side chains of β-(1,6)-d-glucans, with the latter being connected to the inner chains of N-acetyl-glucosamine (forming the chitin fraction) and glycophosphatidylinositol anchors further connected to an outer layer of mannooligosaccharides linked in α-(1,3; 1,4; 1,6) and forming the parietal structure of *Saccharomyces cerevisiae* [35]. This assembly, especially that of β-d-glucans due to its highly organised tridimensional structure consisting of a single helix further associated in a triple helix and forming fibres of glucans, as well as their high degree of polymerisation [36,37], resisted digestion and retained its binding capacity throughout the course of the digestive tract while still maintaining its dynamic properties and preserving cell wall integrity [38]. Biomass density during the fermentation process also plays a role in the resistance of YCW to even complex lytic mixtures [39]. These macromolecules interrelate with mycotoxins, such as AFB1, and sequester them into helical β-d-glucan chains through hydroxyl group hydrogen bonding and van der Waals pi-stacking interactions, as established in previous work [40]. By contrast, inorganic sequestrants, such as HSCAS, rely on their comparatively large cation exchange capacity, significant surface area, and structural channels and pores to facilitate binding interactions (chelation or chemisorption) with mycotoxins such as AFB1 [31]. Differences in physical formations such as channels, pores, and interlayer spaces can restrict the sizes of molecules that can effectively be sequestered. As AFB1 is among the smallest mycotoxin molecules, binding was shown to be quite effective for this toxin, but this does not necessarily hold true for other (larger) mycotoxins [41]. Furthermore, the very inflexible structure of HSCAS compared to the dynamic organisation and adsorption behaviour of YCW, as characterised by the latter’s cooperative and interaction nature, enables YCW to target a larger subset of mycotoxins and generate interactions with zearalenone, deoxynivalenol, patulin [40], and ochratoxin A [28].

Moving into the ex vivo investigations with the third model tested, AFB1 bioavailability was further evaluated. The observed increase in AFB1 uptake in the intestinal tissue following the use of either 0.1% YCW or HSCAS was attributable to the adhesion of the (sorbent + AFB1) complex directly to the surface of the mucosal layer of the intestinal slices used in our study. Further experiments indicated that the increased inclusion levels (1.0% and 5.0%, *w*/*v*) of both adsorbents tended to reduce AFB1 uptake due to an increase in the ratio of the adsorbent over AFB1 (fixed) in addition to the fixed ratio of AFB1 to the mucosal surface of the intestinal slices. The adherence phenomenon did not indicate an increase in the transport of AFB1 through the epithelial membrane of the intestinal tissue, which was confirmed by the results obtained with the fourth model. As indicated, a significant factor in the sequestration of AFB1 was the net negative charge exhibited by HSCAS [31] and the non-charged parietal element of YCW [42] as well as the interactions that could be promoted with the polar uncharged AFB1 molecule [40,43]. Intestinal mucus is produced as a defense mechanism against invasion from intestinal microbiota. The mucin constituents similarly have a net negative charge that acts to draw cations towards itself, thereby facilitating the uptake of many nutrients [44]. The interactions of significant fields of charge with intermediary, positively charged molecules (possibly dissociated elements of the elution buffer) could account for the rapid and forceful degree to which these adsorbent particles adhere to the epithelial mucus. This model indicates that at low AFB1 concentrations in simulated lower GI conditions, differences in the adsorption profiles of YCW and HSCAS are negligible. It is important to note that the interactions between sorbents (containing bound mycotoxin) and gut epithelial tissue may manifest differently in vivo. This interaction may influence commensal microbiota, the immunology of the host organism, or other physiological parameters, such as mucus exudation. It is widely appreciated that mycotoxins can have deleterious effects on bacterial populations much in the same way as they cause damage to eukaryotic cells. Indeed, penicillin is a fungal metabolite and could be considered a mycotoxin [45]. The desorption of toxins from the adsorbent in the vicinity of the gut epithelia could conceivably disrupt commensal microbiota directly or indirectly through the host inflammatory response, potentially promoting pathogenic strains [46]; however, “normal” microbiota typically act to acidify their local environments, thus producing acetate, lactate, butyrate, propionate, and other short chain fatty acids among other bioprotectants [47]. Thus, within the intestinal lumen, particularly at the mucus interface, environmental conditions can enhance the pH-mediated binding of toxins with their sequestrants.

While Model 3 allowed us to determinate the YCW/HSCAS interactions with live rat intestinal tissue in the presence of a low (10 µg/L) concentration of AFB1, it did not allow us to clearly understand the absorption mechanism of the toxin by the same intestinal tissue and its transfer to the apical side and further into systemic circulation. This model also suffered from important variability due to its ex vivo nature and through the time course exposure to the toxin, notably only enabling a 4 min evaluation over a short period of the interaction. In this context, the fourth model focused on a bicameral system (an Ussing chamber) that allowed the effective measurement of AFB1 transport across live intestinal tissue and was able to “complete the journey” of AFB1 through the digestive tract. Phase separation mitigated the issues observed with Model 3, as particle interactions with the membrane in the apical chamber did not inherently lead to measurement complexities in the basolateral chamber. This model clearly demonstrated a significant reduction in the transfer rate of AFB1 into the basolateral chamber for both tested products. The linearity of AFB1 binding by both sequestrants was notable (Figure 5). Increased incubation time (synonymous with gut residence time) lends itself to a more efficacious reduction in AFB1 bioavailability, thereby maximising the protective effect afforded by adsorbents in the feed allocation. Furthermore, the control values better fit second order polynomial kinetics, indicating the progressive acceleration of AFB1 absorption over the course of the experiment; this rate increase is of interest. Evidence from Gallo 2008 [6] that AFB1 is absorbed through a variety of mucosal membranes (and not absorbed specifically in the gut) indicates passive diffusion to be the most likely uptake mechanism, unlike for other mycotoxins, such as deoxynivalenol or ochratoxin A [48]. As AFB1 is known to induce apoptosis in eukaryotic cells [49], and prokaryotic populations have been shown to be negatively affected by AFB1 in a dose-dependent manner [46], it is conceivable that epithelial membrane permeability increased in the presence of 10 µg/L AFB1 due to cellular disruption and, perhaps, the early stage induction of apoptosis. This could also explain the observed acceleration in absorption.

The lack of differences in the accumulation of AFB1 with or without the use of an adsorbent seem to indicate the limited carrying capacity of the intestinal tissue. Contrary to the indications of Model 3, the Model 4 did not record the same adsorbent particle–gut tissue interactions. In the apical solution, the concentration of AFB1 was 30 µg/L, 1000-fold higher than that in the tissue. Moreover, throughout the experiment, the concentration of AFB1 was about 1000-fold higher in the apical than in the basolateral medium. These results suggest that AFB1 did not accumulate in the tissue and that in this system, the rate of AFB1 transport through the rat intestinal tissue was quite slow. In the live rat, AFB1 was mainly transported in the albumin fraction of the blood serum [50]. In the present trial, this biological transport system did not exist. This may partly explain the slow rate of AFB1 transport in the Ussing chamber system. However, the adsorbent concentrations tested in this study were notably increased in Model 4, from 0.1% in earlier models to 0.3%, thus striking a balance between the results from earlier datasets and reflecting the recommended inclusion rates; this result is also consistent with the conventional 0.2% inclusion level [41].

The overall results depicted the applicability of the YCW and HSCAS at interacting effectively with AFB1. The efficacy of the sequestration can however change according to the toxin, YCW having demonstrated significantly higher mitigation activity toward zearalenone compared to HSCAS when investigated in a previous study using the same in vitro and ex vivo evaluation [25]. These materials could thus be of practical interest and applicability in agriculture for mitigating the mycotoxin impact in animals. Mycotoxin occurrence, variety and multiplicity in feedstuffs and corresponding sorbent efficacy should however be considered. Regulatory authorities have approved the use of HSCAS [51] and a comprehensive toxicological and safety evaluation demonstrated no concerns with consumption of YCW products [52], making them suitable for animal use.

## 4. Conclusions

The in vitro evaluation of YCW and HSCAS demonstrated the capacity of both adsorbents to effectively interact with AFB1, as demonstrated in Models 1 and 2. Under acid to neutral pH changes, the YCW material demonstrated an advantage in maintaining effective interaction with AFB1 compared to HSCAS, whose interactions were favoured by acidic conditions. HSCAS, however, exhibited higher sequestration capacity toward AFB1 in the tested digestive conditions compared with YCW. In the intestine, as evaluated using ex vivo Models 3 and 4, YCW and HSCAS were able to significantly reduce the bioavailability of AFB1 and its uptake by intestinal tissue. No significant difference in efficacy was seen between the two tested materials. To summarize, this study showed that the dietary inclusion of YCW or HSCAS could represent a valuable mitigation strategy by limiting the absorption of AFB1 in an animal’s digestive tract.

## 5. Materials and Methods

### 5.1. Model 1: In Vitro (FEFANA) Protocol for Assessing AFB1 Sequestration

The FEFANA guidance document for determining the efficacy of mycotoxin inactivators formed the basis of this experiment [24], following the concentrations established in Yiannikouris et al. [25], with some adjustment due to the solubility of AFB1. Aflatoxin B1 was tested at six concentrations (10, 20, 30, 40, 50, and 60 µg/mL) over three replicates. An AFB1 (Fermentek, Jerusalem, Israel) stock solution was prepared in acetonitrile and diluted appropriately to obtain a stock concentration of 1.0 mg/mL. The accurate concentration of this stock solution was determined by measuring the absorbance of a 1:100 dilution via spectrophotometry at a wavelength of 350 nm and applying the following equation:

AFB1 (µg/mL) = *A* ×MW × 1000)/ (1)

where the absorbance (*A*) at 354 nm is the mean of six replicates, and the molecular weight (MW) and the molecular absorptivity (ε) are 312.3 g/mol and 20,900, respectively. Calibration curves were prepared for HPLC-FLD (high-pressure liquid chromatography with fluorescence detection) responses using the calculated AFB1 concentrations.

Treatments consisted of a control blank (water), yeast cell wall (YCW) of enzymatically hydrolysed *Saccharomyces cerevisiae* sp. *cerevisiae* (test product; Mycosorb^®^, Alltech Inc., Nicholasville, KY, USA), hydrated sodium calcium aluminosilicate (HSCAS) (test product containing ≥70 % smectite (dioctahedral montmorillonite); supplied by Alltech Inc., Nicholasville, KY, USA), or activated carbon (AC) (reference material; Norit CASP, Sigma-Aldrich, St. Louis, MO, USA). Treatments were prepared by pipetting 200 µL of the appropriate binding material suspension (10 mg/mL) into test tubes to obtain 0.1% *w*/*v* concentrations, the minimum administration dosage according to the supplier (Alltech) guidelines. All suspensions were mixed continuously to ensure homogeneity during pipetting. A 1600 µL volume of either phosphate buffer (pH 7.0) or citrate buffer (pH 3.0) was then added to the test tubes containing the different treatments. Acetonitrile and AFB1 stock solution (1 mg/mL), in this exact order, were added to achieve the final AFB1 10–60 µg/mL concentrations. The test tubes were then continuously mixed during the 90 min incubation at 37 °C. Following centrifugation (3000× *g* for 10 min), the supernatant was decanted into HPLC vials and placed in the autosampler for further HPLC analysis.

HPLC-FLD analysis was carried out on the samples using a Kinetex C18 2.6 µm (50 mm × 2.1 mm) reverse phase column (Phenomenex, Torrance, CA, USA), and a methanol/water mixture (35:65, *v*/*v*) was used for isocratic elution. The flow rate of the mobile phase was adjusted to 0.4 mL/min, with a 5 µL injection volume over a 6 min run time. AFB1 fluorescence quantification was performed at a 365 nm excitation wavelength and a 435 nm emission wavelength.

The Freundlich equation [53] was applied to analyse the experimental data:

*C*_ads_ = *K*_F_*C*_aq_^1/*n*^ (2)

where *C*_ads_ is the concentration adsorbed (µg/g); *C*aq is the equilibrium concentration in the solution (µg/mL); *K*_F_ is the Freundlich adsorption capacity at unit concentration (µg/g); and 1/*n* is the Freundlich adsorption intensity.

### 5.2. Model 2: In Vitro Assessment of AFB1 Binding in Simulated Stomach Conditions

This experiment followed a similar protocol for Model 2 as per Yiannikouris et al. [25]; however, some modifications were implemented. Tests were carried out in 20 mL silanised glass liquid scintillation vials in a 15 mL reaction volume. Here, 1, 10, or 50 mg portions of test material (YCW or HSCAS) and 1 g of feed (Alimetrics Ltd., Espoo, Finland) were suspended in water in triplicates. The experiment commenced with the addition of ^3^H-labelled AFB1 (Moravek Biochemicals Inc., Brea, CA, USA) at 10 µg/L, giving activity of 0.005 µCi mL^−1^. The mixtures were incubated for 2 h at 37 °C with gentle shaking. Following incubation, the tubes were centrifuged at 3000× *g* for 15 min, separating the test materials from the aqueous phase. A volume of 500 µL of supernatant was collected from the reaction vessels and mixed with a 3 mL scintillation cocktail (Ultima Gold, Perkin Elmer, Waltham, MA, USA) in plastic 3.5 mL liquid scintillation tubes before being analysed for tritium activity using a liquid scintillation counter (1450 MicroBeta TriLux, Perkin Elmer, MA, USA). After the first samples (before pepsin–HCl treatment) were taken, the mixtures were resuspended and homogenised, treated with 4.5 mL of 150 mM HCl plus 1.5 mL of activated pepsin solution, and incubated for a further 2 h at 37 °C with gentle shaking. Following this incubation, the tubes were again centrifuged at 3000× *g* for 15 min, and 500 µL of the supernatant was drawn off and analysed as above.

### 5.3. Model 3: Ex Vivo Demonstration of Binding Efficiency in the Lower Gastro-Intestinal Tract

Ten 7-week-old Han/Wistar rats were utilised for this experiment (~250 g body weight). The rats were fed with a Breeding Diet 1314 Forti (Altromin, Lage, Germany) and given ad libitum access to feed and water until the time of the experiment. The rats had no exposure to aflatoxin prior to this experiment. Euthanisation was performed with CO_2_ asphyxiation followed by dislocation of the cervical vertebrae. A medial incision was immediately made on the peritoneal cavity post-mortem to allow excision of the proximal jejunum. This region of the small intestine (along the attachment of the mesentery) was then opened longitudinally. The digesta was gently removed, and the tissue was rinsed with ice-cold, isotonic saline solution. The live intestinal tissue was cut into 2 mm slices with a mean weight of 27 mg. Sixteen slices were prepared from each rat. The slices were kept in a carbogen-gassed (95% O_2_, 5% CO_2_) ice-cold isotonic saline solution before being transferred to warm (37 °C) medium a few minutes before the incubation treatment. Intestinal slices underwent treatment within 45 min of the animal’s death to ensure maintenance of their viability.

A pH 7.3 isotonic saline solution was prepared, as follows, for all rinsing and incubation processes: 128 mmol/L NaCl, 4.7 mmol/L KCl, 2.5 mmol/L CaCl_2_, 1.2 mmol/L KH_2_PO_4_, 2.6 mmol/L MgSO_4_, and 2.0 mmol/L NaHCO_3_. A solution of 5 mmol/L d-glucose was also included in the storage medium; however, treatments were conducted in a glucose-free solution.

^3^H-labelled AFB1 (Moravek Biochemicals Inc., Brea, CA, USA) was spiked into the AFB1 stock solution to achieve 0.15 µCi/mL, and the concentration of AFB1 was set to 10 µg/L for this experiment. Tissue slices were treated in duplicate as follows: the inclusion of YCW at 1 or 10 mg/mL, or HSCAS at 10 mg/mL. Control treatments were identical except for exclusion of the test materials. Tissue slices were gently shaken during incubation in 2 mL volumes of the test medium in glass vials maintained at 37 °C for 0 (a 1 s dip in the treatment solution), 1, 2, and 4 min, and in a pre-study experiment up to 8 min, or before being rinsed in an ice-cold isotonic saline solution to remove the ^3^H-labelled incubation medium from the tissue surface. Tissue paper was used to remove excess rinse solution from the tissue slice before being placed into pre-weighed 3 mL plastic liquid scintillation vials. The vials were weighed again to assess the tissue mass, and the slices were solubilised overnight in a tissue solvent (Solvable, Perkin Elmer, Waltham, MA, USA). Three millilitres of the liquid scintillation cocktail (Ultima Gold, Perkin Elmer, Waltham, MA, USA) was added, and the radioactivity of the samples was measured by liquid scintillation counting.

The amount of absorbed AFB1 was determined from the radioactivity detected in the samples, with the assumption that all the measured activity came from the ^3^H-labelled AFB1. The results were corrected for quenching by the tissue and tissue solvent and the efficacy of liquid scintillation counting for tritium under the used conditions.

### 5.4. Model 4: Impact of Adsorption Capacity on Transmission of AFB1 through Live Intestinal Tissue

Ten eight-week-old male Han/Wistar rats (mean weight of 295 g) were used for this study. The rats were fed with the Breeding Diet 1314 Forti (Altromin, Lage, Germany) and given ad libitum access to feed and water until the time of the experiment. The rats were not exposed to aflatoxin prior to the experiment. As per Model 3, the rats were euthanised with CO_2_ asphyxiation and cervical dislocation. Immediately after the death of the animal, the abdominal cavity was opened, and the proximal jejunum was removed and opened longitudinally along the attachment of the mesentery. The digesta was gently removed, and the tissue was rinsed with ice-cold, isotonic saline solution. Two-centimeter pieces of the intestinal tissue were cut and pinned between the apical and basolateral halves of the Ussing chambers.

An isotonic saline solution at pH 7.3 was prepared, as follows, for all rinsing and incubation processes: 128 mmol/L NaCl, 4.7 mmol/L KCl, 2.5 mmol/L CaCl_2_, 1.2 mmol/L KH_2_PO_4_, 2.6 mmol/L MgSO_4_, 2.0 mmol/L NaHCO_3_, and 5 mmol/L d-glucose. Ethanol was added at a level of 1.5% to increase the solubility of AFB1.

AFB1 and the test substances were only added to the apical chambers of the Ussing apparatus. As in [25], the total AFB1 concentration in the apical medium was 30 µg/L, and activity of the ^3^H-labelled AFB1 was 0.5 µCi/mL.

The tissue slices were treated with the test substances (YCW or HSCAS) at 3 mg/mL for their respective treatments. No adsorbent was added to the control treatment. The incubation media were prepared a minimum of 1 h before each experiment to allow AFB1 to bind to the test substances. Both the Ussing chamber system and the incubation media were pre-warmed to 37 °C before the experiment. After pinning the intestinal slice between the chambers, 3.5 mL of apical and basolateral media were added to the respective chambers. The medium in the chambers was circulated and oxygenated via carbogen (95% O_2_, 5% CO_2_) bubbling through the chamber system.

Samples were then taken at the beginning and the end of the experiment from the apical chamber, and 40 µL volumes were taken from the basolateral chamber at 20, 40, 60, 80, 100, and 120 min time points. Each sample was placed into a liquid scintillation vial containing 3 mL liquid scintillation cocktail (Ultima Gold, Perkin Elmer, Waltham, MA, USA) and r ^3^H activity was measured using a liquid scintillation counter. After the incubation period, the intestinal piece was removed from the chamber. Approximately one-third of each piece was weighed, solubilised in tissue solvent, and measured for tritium activity, as for Model 3.

### 5.5. Statistical Analysis

#### 5.5.1. Model 1

The free AFB1 present after the equilibrium reactions at pH 3.0 and 7.0 was plotted against the bound AFB1 per mass of product for the different adsorbent materials tested and according to the mycotoxin inactivator testing recommendations of FEFANA. In the present work, the three recommended mathematical equations were used to calculate the amount of bound mycotoxin per unit of mass of the test products, and the goodness of fit was tested for each material. The Freundlich model was ultimately chosen as it provided the best fit for the dataset (Microsoft Excel version 2019, Portland, OR, USA). The equation used was as follows: Qeq = Kf Ceq1/n where Qeq is the y-axis, and Ceq is the *x*-axis. Two-factor ANOVA and Tukey’s honestly significant difference (HSD) tests, with pairwise t-tests, were applied to statistically compare the measurements to the controls and other treatments (SPSS, IBM, version 22).

#### 5.5.2. Model 2

In this model, the feed was amended (or not) with either YCW or HSCAS at three inclusion levels each, providing seven experimental treatments in total (control; 0.1%, 1.0%, and 5.0% YCW; 0.1%, 1.0%, and 5.0% HSCAS). These treatments were compared before and after treatment with pepsin–HCl via a one-way ANOVA, with Tukey’s post hoc analysis used to determine the pairwise differences between the treatment groups. Single-factor ANOVA and pairwise *t*-tests were applied to statistically compare these measurements to the controls and the other treatments.

#### 5.5.3. Model 3

For each time point (0, 1, 2 and 4 min), a one-way ANOVA was conducted to determine if there was a difference between the AFB1 uptake for each of the four treatments (Control, HSCAS 0.1%, YCW 0.1% and YCW 1.0%). Tukey’s post hoc analysis was then used to determine any pairwise differences between the treatment groups. Dixon’s Q test was further applied to identify and assess statistical outliers.

#### 5.5.4. Model 4

Data were plotted as the AFB1 uptake in the basolateral compartment against time (mins), and linear and polynomial regression analyses were applied to each variable to determine which provided the best fit. The treatments were compared statistically at 120 min using a one-way ANOVA with Tukey’s post hoc analysis used to determine the pairwise differences between the treatment groups.

## Figures and Tables

**Table 1:** Constants related to the adsorption capacity and intensity toward aflatoxin B1 (AFB1) evaluated at pH 3.0 or 7.0 using either a yeast cell wall-based adsorbent (YCW) or hydrated sodium calcium aluminosilicate (HSCAS) compared with an activated carbon (AC) reference material, calculated from the Freundlich equation and the corresponding coefficient of regression.

Adsorbent	pH	Kf	*n*	*R* ^2^
HSCAS	3.0	5.01	1.51	0.9891
YCW	3.0	5.47	2.66	0.9404
AC	3.0	108.42	2.29	0.6396
HSCAS	7.0	1.54	1.13	0.9712
YCW	7.0	1.37	1.38	0.8638
AC	7.0	136.55	2.28	-

**Table 2:** The proportion of bound aflatoxin B1 (AFB1), as a function of increasing AFB1 levels, added to the reaction environment evaluated at pH 3.0 or 7.0 in the presence of either a yeast cell wall-based adsorbent (YCW) or hydrated sodium calcium aluminosilicate (HSCAS) compared with an activated carbon (AC) reference material.

Initial AFB1 ^1^	pH	Bound AFB1 (%) (Mean ± Standard Error ^2^)		
(µg/mL)		HSCAS	YCW	AC
10	3.0	77.92 ± 2.54	74.34 ± 1.38	100 ± 3.63
20	3.0	74.62 ± 0.39	62.02 ± 3.4	100 ± 0.26
30	3.0	72.05 ± 0.91	55.75 ± 3.62	99.82 ± 0.07
40	3.0	70.16 ± 1.57	47.25 ± 2.01	99.8 ± 0.67
50	3.0	67.16 ± 1.09	41.54 ± 2.65	99.73 ± 0.17
60	3.0	65.02 ± 0.75	38.9 ± 0.83	99.65 ± 0.9
Overall Mean ^3^		71.16 ± 4.57 ^c^	53.31 ± 12.87 ^b^	99.83 ± 0.13 ^a^
10	7.0	52.16 ± 1.57	41.95 ± 0.73	100 ± 0.95
20	7.0	52.15 ± 1.85	34.39 ± 1.63	100 ± 1.72
30	7.0	53.18 ± 1.62	41.02 ± 1.53	99.87 ± 0.45
40	7.0	54.33 ± 1.11	36.43 ± 4.45	99.86 ± 0.6
50	7.0	51.94 ± 2.81	36.47 ± 0.77	99.79 ± 0.65
60	7.0	50.41 ± 1.61	31.74 ± 5.12	100.37 ± 2.05
Overall Mean ^3^		52.42 ± 1.78 ^c^	37.17 ± 4.43 ^b^	99.89 ± 0.1 ^a^

^1^ AFB1 initially added to the reaction media. ^2^ Values are the means of three replicates. ^3^ Two-factor ANOVA followed by Tukey honestly significant difference (HSD) post hoc test (*p* < 0.001). Different superscript letters indicate significant differences between treatments.

**Table 3:** Effect of a feed material on the concentration of free aflatoxin B1 (AFB1, μg/L) and evaluation of the percentage reduction in the AFB1 initially present in the reaction environment when amended with either a yeast cell wall-based adsorbent (YCW) or hydrated sodium calcium aluminosilicate (HSCAS), before and after the application of a digestive treatment (pepsin–HCl).

Tested Material ^1^	Untreated		Mean Value ± Standard Error (µg/L)	Pepsin–HCl Treated (µg/L)
	Mean Value ± Standard Error (µg/L)	Reduction (%)		Reduction (%)
Initial free AFB1	10.0		10.0	
Feed alone	7.25 ± 0.35 ^a^	−27.5	5.14 ± 0.02 ^a^	−48.6
+ YCW (0.1%)	7.00 ± 0.25 ^a^	−30.0	5.04 ± 0.04 ^a^	−49.6
+ YCW (1%)	5.81 ± 0.27 ^b^	−41.9	4.01 ± 0.10 ^c^	−59.9
+ YCW (5%)	2.63 ± 0.16 ^c^	−73.7	2.43 ± 0.07 ^d^	−75.7
+ HSCAS (0.1%)	6.62 ± 0.21 ^ab^	−33.8	4.60 ± 0.13 ^b^	−54.0
+ HSCAS (1%)	2.55 ± 0.08 ^cd^	−74.5	2.33 ± 0.18 ^d^	−76.7
+ HSCAS (5%)	1.43 ± 0.12 ^d^	−85.7	1.54 ± 0.03 ^e^	−84.6

^1^ One-factor ANOVA, Tukey’s post hoc test (*p* < 0.05). Different superscript letters indicate significant difference between treatments for the untreated and pepsin–HCl-treated groups separately.

**Table 4:** Quantification and statistical significance of aflatoxin B1 (AFB1) accumulation on gut epithelial slices over time in the presence of either a yeast cell wall-based adsorbent (YCW) or hydrated sodium calcium aluminosilicate (HSCAS).

Treatments	AFB1 (µmol/g Tissue ± Standard Error ^1^)			
	0 min	1 min	2 min	4 min
Control	0.0085 ± 0.0010 ^a^	0.0529 ± 0.0034 ^a^	0.0629 ± 0.0034 ^a^	0.0896 ± 0.0057 ^a^
HSCAS (0.1%) ^2^	0.0144 ± 0.0015 ^b^	0.1898 ± 0.0168 ^b^	0.2163 ± 0.0228 ^b^	0.2665 ± 0.0261 ^b^
YCW (0.1%) ^2^	0.0140 ±0.0015 ^b^	0.2098 ± 0.0315 ^b^	0.2205 ± 0.0388 ^b^	0.3124 ± 0.0455 ^b^
YCW (1.0%) ^2^	0.0134 ± 0.0016 ^ab^	0.1372 ± 0.0173 ^b^	0.1984 ± 0.0242 ^b^	0.2444 ± 0.0233 ^b^

^1^ Mean values of ten replicates; one-way ANOVA for each incubation time condition, Tukey HSD, Q test (*p* < 0.05). Different superscript letters indicate significant differences between treatments. ^2^ Inclusion rate (%) is the weight of each adsorbent to the volume of reaction media.

**Table 5:** The regression analysis results of the aflatoxin B1 (AFB1, µmol/g of tissue/min) accumulation on intestinal tissue slices with or without (Control) the addition of either a yeast cell wall-based adsorbent (YCW) or hydrated sodium calcium aluminosilicate (HSCAS).

Treatments		Coefficient	S.E.	*t* Stat	*p*-Value
Control	Slope	0.0124	0.0020	6.3260	7.6 × 10^−7^
	Intercept	0.0395	0.0052	7.624	2.6 × 10^−8^
HSCAS (0.1%) ^1^	Slope	0.0255	0.0101	2.5267	1.7 × 10^−2^
	Intercept	0.1647	0.0267	6.1659	1.2 × 10^−6^
YCW (0.1%) ^1^	Slope	0.0359	0.0178	2.0135	5.4 × 10^−2^
	Intercept	0.1638	0.0472	3.4744	1.7 × 10^−3^
YCW (1.0%) ^1^	Slope	0.0339	0.0101	3.3712	2.2 × 10^−3^
	Intercept	0.1141	0.0266	4.2846	2.0 × 10^−4^

^1^ Inclusion rate (%) is the weight of each adsorbent to the volume of reaction media.

**Table 6:** Regression analysis comparison between aflatoxin B1 (AFB1 uptake in ng/L/min) treatment alone (Control) and in combination with either a yeast cell wall-based adsorbent (YCW) or hydrated sodium calcium aluminosilicate (HSCAS) (0.3%) over an absorption time of 120 min in an Ussing chamber ex vivo system using first order polynomial (linear) modelling for kinetics of each of the treatments.

Treatments		Coefficient	*p*-Value	95% Conf. Interval	
**Control**	Slope	2.39	0.00	1.98	2.80
	Intercept	−24.61	0.12	−55.88	6.66
YCW (0.3%) ^1^	Slope	0.71	0.00	0.33	1.09
	Intercept	28.78	0.06	−0.69	58.24
HSCAS (0.3%) ^1^	Slope	0.20	0.07	−0.01	0.41
	Intercept	40.89	0.00	24.35	57.43

^1^ Inclusion rate (%) is the weight of each adsorbent to the volume of reaction media.

## References

[1] Frisvad J.C., Hubka V., Ezekiel C.N., Hong S.B., Nováková A., Chen A.J., Arzanlou M., Larsen T.O., Sklenář F., Mahakarnchanakul W. (2019). Taxonomy of Aspergillus section Flavi and their production of aflatoxins, ochratoxins and other mycotoxins. Stud. Mycol..

[2] Asao T., Büchi G., Abdel-Kader M.M., Chang S.B., Wick E.L., Wogan G.N. (1963). Aflatoxins B and G. J. Am. Chem. Soc..

[3] Pitt J.I. (2000). Toxigenic fungi and mycotoxins. Br. Med. Bull..

[4] Rushing B.R., Selim M.I. (2019). Aflatoxin B1: A review on metabolism, toxicity, occurrence in food, occupational exposure, and detoxification methods. Food Chem. Toxicol..

[5] Ramos A.J., Hernández E. (1996). In situ absorption of aflatoxins in rat small intestine. Mycopathologia.

[6] Gallo A., Moschini M., Masoero F. (2008). Aflatoxins absorption in the gastro-intestinal tract and in the vaginal mucosa in lactating dairy cows. Ital. J. Anim. Sci..

[7] Deng J., Zhao L., Zhang N.Y., Karrow N.A., Krumm C.S., Qi D.S., Sun L.H. (2018). Aflatoxin B 1 metabolism: Regulation by phase I and II metabolizing enzymes and chemoprotective agents. Mutat. Res. Rev. Mutat. Res..

[8] Wogan G.N., Kensler T.W., Groopman J.D. (2012). Present and future directions of translational research on aflatoxin and hepatocellular carcinoma. A review. Food Addit. Contam. Part A Chem. Anal. Control. Expo. Risk Assess..

[9] Rotimi O.A., Rotimi S.O., Goodrich J.M., Adelani I.B., Agbonihale E., Talabi G. (2019). Time-course effects of acute aflatoxin B1 exposure on hepatic mitochondrial lipids and oxidative stress in rats. Front. Pharmacol..

[10] Arenas-Huertero F., Zaragoza-Ojeda M., Sánchez-Alarcón J., Milić M., Klarić M.Š., Montiel-González J.M., Valencia-Quintana R. (2019). Involvement of Ahr Pathway in Toxicity of Aflatoxins and Other Mycotoxins. Front. Microbiol..

[11] Kujawa M. (1993). Some Naturally Occurring Substances: Food Items and Constituents, Heterocyclic Aromatic Amines and Mycotoxins. IARC Monographs on the Evaluation of Carcinogenic Risk of Chemicals to Humans.

[12] Massey T.E., Stewart R.K., Daniels J.M., Liu L. (1995). Biochemical and Molecular Aspects of Mammalian Susceptibility to Aflatoxin B1 Carcinogenicity. Proc. Soc. Exp. Biol. Med..

[13] Kumar P., Mahato D.K., Kamle M., Mohanta T.K., Kang S.G. (2017). Aflatoxins: A global concern for food safety, human health and their management. Front. Microbiol..

[14] (2000). Guidance for Industry: Action Levels for Poisonous or Deleterious Substances in Human Food and Animal Feed | FDA, U.S. Food Drug Adm. *Cent. Food Saf. Appl. Nutr*. https://www.fda.gov/regulatory-information/search-fda-guidance-documents/guidance-industry-action-levels-poisonous-or-deleterious-substances-human-food-and-animal-feed.

[15] European Commission (2002). Directive 2002/32/EC of the European Parliament and of the European Council of May 7^th^ 2002 on undesirable substances in animal feed-Council statement. Off. J. Eur. Union..

[16] Jouany J.P. (2007). Methods for preventing, decontaminating and minimizing the toxicity of mycotoxins in feeds. Anim. Feed Sci. Technol..

[17] Moretti A., Pascale M., Logrieco A.F. (2019). Mycotoxin risks under a climate change scenario in Europe. Trends Food Sci. Technol..

[18] Whitaker T.B., Slate A.B., Johansson A.S., Diaz D., Mycotoxin Blue B. (2005). Sampling Feeds for Mycotoxin Analysis.

[19] (2006). Commission Regulation (EC) No 401/2006 of 23 February 2006 laying down the methods of sampling and analysis for the official control of the levels of mycotoxins in foodstuffs. Off. J. Eur. Union..

[20] Niderkorn V., Boudra H., Morgavi D. (2007). Les fusariotoxines: Comment limiter leur présence dans les ensilages et leur impact chez les ruminants?. Fourrag.

[21] Firmin S., Gandia P., Morgavi D.P., Houin G., Jouany J.P., Bertin G., Boudra H. (2010). Modification of aflatoxin B1 and ochratoxin a toxicokinetics in rats administered a yeast cell wall preparation. Food Addit. Contam. Part A Chem. Anal. Control. Expo. Risk Assess..

[22] Firmin S., Morgavi D.P., Yiannikouris A., Boudra H. (2011). Effectiveness of modified yeast cell wall extracts to reduce aflatoxin B1 absorption in dairy ewes. J. Dairy Sci..

[23] Kim S.W., Holanda D.M., Gao X., Park I., Yiannikouris A. (2019). Efficacy of a yeast cell wall extract to mitigate the effect of naturally co-occurring mycotoxins contaminating feed ingredients fed to young pigs: Impact on gut health, microbiome, and growth. Toxins.

[24] FEFANA (2009). Animal feeding-stuffs–Determination of in vitro efficacy of Mycotoxin Inactivators based on adsorption assay of Aflatoxin B1. In *DRAFT Mycotoxin Inactivators: Adsorption Method*.

[25] Yiannikouris A., Kettunen H., Apajalahti J., Pennala E., Moran C.A. (2013). Comparison of the sequestering properties of yeast cell wall extract and hydrated sodium calcium aluminosilicate in three in vitro models accounting for the animal physiological bioavailability of zearalenone. Food Addit. Contam. Part A Chem. Anal. Control. Expo. Risk Assess..

[26] Yiannikouris A., Poughon L., Cameleyre X., Dussap C., Jouany J., François J. (2003). A novel technique to evaluate interactions between Saccharomyces cerevisiae cell wall and mycotoxins: Application to zearalenone. Biotechnol. Lett..

[27] Yiannikouris A., André G., Buléon A., Jeminet G., Canet I., François J., Bertin G., Jouany J.P. (2004). Comprehensive conformational study of key interactions involved in zearalenone complexation with β-D-glucans. Biomacromolecules.

[28] Vartiainen S., Yiannikouris A., Apajalahti J., Moran C.A. (2020). Comprehensive evaluation of the efficiency of yeast cell wall extract to adsorb ochratoxin A and mitigate accumulation of the toxin in broiler chickens. Toxins.

[29] Fochesato A.S., Cuello D., Poloni V., Galvagno M.A., Dogi C.A., Cavaglieri L.R. (2019). Aflatoxin B1 adsorption/desorption dynamics in the presence of Lactobacillus rhamnosus RC007 in a gastrointestinal tract-simulated model. J. Appl. Microbiol..

[30] Li J.J., Suo D.C., Su X.O. (2010). Binding capacity for aflatoxin B1 by different adsorbents. Agric. Sci. China.

[31] Jaynes W.F., Zartman R.E., Hudnall W.H. (2007). Aflatoxin B1 adsorption by clays from water and corn meal. Appl. Clay Sci..

[32] Tangni E.K., de Rouck G., Potel A., de Meeûs L., Aerts G., Larondelle Y. Towards Mycotoxin Control in Brewing: Adfimax^®^ as a novel promising solution to overcome this challenge. Proceedings of the 30th International Congress European Brewery Convention.

[33] Frassoldati E.B.T.-R.M., Ranzi C. (2019). Molecular Sciences and Chemical Engineering, Modeling of Thermochemical Conversion of Biomasses.

[34] Tiwari U.P., Singh A.K., Jha R. (2019). Fermentation characteristics of resistant starch, arabinoxylan, and β-glucan and their effects on the gut microbial ecology of pigs: A review. Anim. Nutr..

[35] Cabib E., Roh D.H., Schmidt M., Crotti L.B., Varma A. (2001). The yeast cell wall and septum as paradigms of cell growth and morphogenesis. J. Biol. Chem..

[36] Aguilar-Uscanga B., François J.M. (2003). A study of the yeast cell wall composition and structure in response to growth conditions and mode of cultivation. Lett. Appl. Microbiol..

[37] Sletmoen M., Stokke B.T. (2008). Higher order structure of (1,3)-β-D-glucans and its influence on their biological activities and complexation abilities. Biopolymers.

[38] Klis F.M., Mol P., Hellingwerf K., Brul S. (2002). Dynamics of cell wall structure in Saccharomyces cerevisiae. FEMS Microbiol. Rev..

[39] Ovalle R., Lim S.T., Holder B., Jue C.K., Moore C.W., Lipke P.N. (1998). A spheroplast rate assay for determination of cell wall integrity in yeast. Yeast.

[40] Yiannikouris A., André G., Poughon L., François J., Dussap C., Jeminet G., Bertin G., Jouany J.-P. (2006). Chemical and Conformational Study of the Interactions Involved in Mycotoxin Complexation with β-D-Glucans. Biomacromolecules.

[41] Kolawole O., Meneely J., Greer B., Chevallier O., Jones D.S., Connolly L., Elliott C. (2019). Comparative in vitro assessment of a range of commercial feed additives with multiple mycotoxin binding claims. Toxins.

[42] Lipke P.N., Ovalle R. (1998). Cell wall architecture in yeast: New structure and new challenges. J. Bacteriol..

[43] Ramales-Valderrama R., Vázquez-Durán A., Méndez-Albores A. (2016). Biosorption of B-aflatoxins using biomasses obtained from formosa firethorn [Pyracantha koidzumii (Hayata) Rehder]. Toxins.

[44] Leal J., Smyth H.D.C., Gosh D. (2018). Physicochemical properties of mucus and their impact on transmucosal drug delivery. Int. J. Pharm..

[45] Speight N., Kohlstadt I. (2012). Mycotoxin-Related Illness.

[46] Wang J., Tang L., Glenn T.C., Wang J.S. (2016). Aflatoxin B1 induced compositional changes in gut microbial communities of male F344 rats. Toxicol. Sci..

[47] Iacob S., Iacob D.G., Luminos L.M. (2019). Intestinal microbiota as a host defense mechanism to infectious threats. Front. Microbiol..

[48] Sergent T., Ribonnet L., Kolosova A., Garsou S., Schaut A., de Saeger S., van Peteghem C., Larondelle Y., Pussemier L., Schneider Y.J. (2008). Molecular and cellular effects of food contaminants and secondary plant components and their plausible interactions at the intestinal level. Food Chem. Toxicol..

[49] Zheng Z., Zuo Z., Zhu P., Wang F., Yin H., Peng X., Fang J., Cui H., Gao C., Song H. (2017). A study on the expression of apoptotic molecules related to death receptor and endoplasmic reticulum pathways in the jejunum of AFB1-intoxicated chickens. Oncotarget.

[50] Dirr H.W., Schabort J.C. (1986). Afaltoxin B1 transport in rat blood plasma. Binding to albumin in vivo and in vitro and spectrofluorimetric studies into the nature of the interaction. Biochim. Biophys. Acta Gen. Subj..

[51] European Commission (2013). Commission implementing Regulation (EU) No 1060/2013 of 29 October 2013 concerning the authorisation of bentonite as a feed additive for all animal species. Off. J. Eur. Union.

[52] Dillon G.P., Yiannikouris A., Moran C.A. (2021). Toxicological evaluation of a glycan preparation from an enzymatic hydrolysis of Saccharomyces cerevisiae. Food Chem. Tox..

[53] Freundlich H. (1907). Über die Adsorption in Lösungen [On adsorption in solutions]. Z. Phys. Chem..

